# Comparison of the effects between arthroscopic transtibial pullout technique and all-inside repair in the treatment of medial meniscus posterior root tears

**DOI:** 10.1016/j.jor.2024.12.003

**Published:** 2024-12-16

**Authors:** Jun Li, Pengfei Shen, Tao Zou, Wen Min, Yuxing Qu, Zikang Xie, Chengjian Wei

**Affiliations:** a Changzhou TCM Hospital Affiliated to Nanjing University of Chinese Medicine, Department of Orthopedics, Changzhou, 213000, PR China; b Jiangsu Province Hospital of Traditional Chinese Medicine, Department of Orthopedics, Nanjing, 210000, PR China

**Keywords:** Medial meniscus, Posterior root tear, Transtibial pullout, All-inside repair

## Abstract

**Background:**

Medial meniscus posterior root tears (MMPRTs) significantly contribute to knee dysfunction, leading to abnormal biomechanics and accelerated cartilage degeneration. Arthroscopic transtibial pullout and all-inside repair are two commonly used techniques for treating MMPRTs, each with unique advantages and limitations.

**Objective:**

To compare the clinical and functional outcomes of the transtibial pullout and all-inside repair techniques in the treatment of MMPRTs, with a focus on postoperative recovery, knee function, and complications.

**Methods:**

40 patients with MMPRTs were randomized to undergo either the transtibial pullout or all-inside repair technique. Clinical outcomes were evaluated using the International Knee Documentation Committee (IKDC) score, Tegner activity scale, Lysholm score, and active range of motion (AROM) of knee flexion, both before and after surgery. Data on operative time, time to ambulation, hospital stay duration, and complications were also collected.

**Results:**

Both surgical groups showed significant improvements in clinical outcomes postoperatively (p < 0.001). The transtibial group exhibited greater functional recovery, with IKDC, Tegner, and Lysholm scores improving by approximately 60 %, 110 %, and 68 %, respectively, compared to the all-inside group. However, complications were more frequent in the transtibial group, including three cases of wound healing issues and one infection, while the all-inside group had one case of deep vein thrombosis. No re-tears were observed in either group during follow-up.

**Conclusion:**

Both the transtibial pullout and all-inside repair techniques effectively restore knee function in patients with MMPRTs. While the transtibial pullout provides better functional outcomes, it is associated with a higher complication rate. The choice of surgical approach should consider patient-specific factors, including tear characteristics and overall health, to optimize results.

## 1 Introduction

**Fig. 1:**
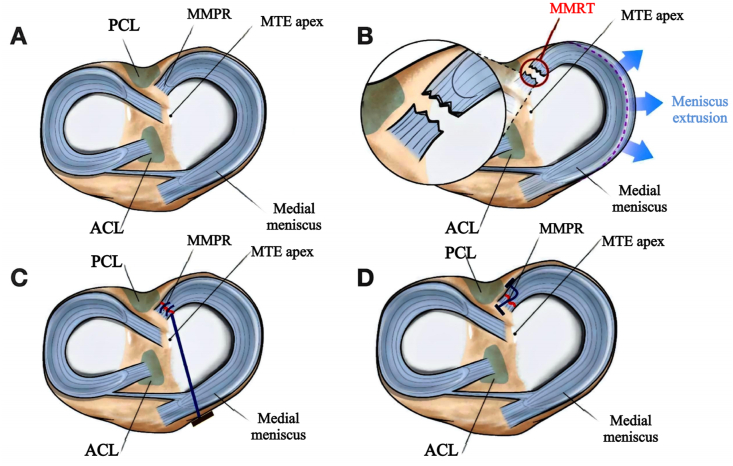
This figure presents the standard knee joint anatomy, the damage to the medial meniscus posterior root, and the Transtibial Pullout technique and All-inside repair, illustrating structures such as the ACL, PCL, MMPR and MTEapex. (A) Presents the standard anatomy of the knee joint, including the femur, tibia, and patella, along with surrounding soft tissues and ligaments. The medial meniscus is located on the medial side of the tibial plateau, with a normal C-shape, showing no signs of fissures, tears, or degeneration. The joint space is clearly visible, and the cartilage surface is smooth and continuous without any signs of abnormal wear or pathological changes, indicating normal knee function; (B) shows the damage to the posterior root of the medial meniscus. The structural integrity between the posterior root and the tibial attachment is disrupted, with partial or complete tearing, causing the meniscus to shift medially or collapse; (C) *Transtibial Pullout technique.*That depicts the condition of the meniscus posterior root following tibial tunnel reconstruction. A tibial tunnel is clearly visible, drilled through the tibia to guide the suture for attachment to the posterior root of the meniscus; (D) *All-inside repair technique*.That shows the arthroscopic repair of the posterior root of the medial meniscus. The suture is clearly visible, guided through the posterior root tissue of the meniscus under arthroscopic visualization, and anchored to the posterior part of the meniscus and the joint capsule. *ACL, Anterior Cruciate Ligament; PCL, Posterior Cruciate Ligament; MMPR, Medial Meniscus Posterior Root; MTE apex, Medial Tibial Eminence apex; MMRT, Medial Meniscus Root Tear.*

Medial meniscal posterior root tears (MMPRTs) are a significant cause of knee joint dysfunction, leading to altered biomechanics and accelerated cartilage degeneration if left untreated.^1^ The posterior root of the meniscus is critical for distributing load across the knee and maintaining joint stability.^2^ A tear in this region compromises the meniscus's ability to function properly, resulting in increased stress on the articular cartilage and a higher risk of osteoarthritis.^3^^,^^4^ Therefore, timely and effective repair of meniscal root tears is essential to restore knee function and prevent long-term joint deterioration.^3^ (See Fig. 1)

Two primary arthroscopic techniques are commonly used to repair meniscal posterior root tears: the transtibial pullout technique and the all-inside repair technique.^5^ Among these, the transtibial pullout technique has gained recognition for its superior biomechanical stability.^6^ By creating a tibial tunnel and securing the torn meniscal root at its anatomic insertion, this technique restores the load-bearing capacity of the meniscus more effectively.^5^ Studies have demonstrated that the transtibial technique provides a stronger fixation and superior long-term stability, which is crucial for maintaining the repaired meniscus's function and reducing the risk of repair failure or re-tear.^7^^,^^8^ The tibial tunnel also allows for more controlled and precise tensioning, leading to more reliable clinical outcomes.^8^

In contrast, the all-inside repair technique offers a less invasive approach by avoiding the creation of a tibial tunnel and using specialized devices to secure the torn meniscus.^9^ While this method has the advantage of reducing surgical trauma, concerns remain regarding its ability to achieve the same level of biomechanical stability as the transtibial pullout technique.^10^ The fixation strength of the all-inside technique may be insufficient for more complex root tears, potentially leading to higher failure rates or suboptimal postoperative outcomes.^11^

This study aims to compare the clinical and functional outcomes between the arthroscopic transtibial pullout technique and the all-inside repair technique in the treatment of meniscal posterior root tears. Special emphasis will be placed on evaluating the superior stability offered by the transtibial pullout technique and its potential advantages in achieving better long-term results. By analyzing postoperative recovery, joint function, and complication rates, this research seeks to provide valuable insights for clinicians in selecting the most appropriate surgical approach for patients with meniscal root tears.

## 2 Materials and methods

### 2.1 Study design

This study was approved by the Medical Ethics Committee. All patient information is stored in the hospital database for research purposes. Inclusion criteria: 1. Diagnosed with medial meniscus posterior root tear by MRI examination, accompanied by corresponding clinical symptoms; 2. Patients over 18 years old with posterior root tear of the meniscus or radial tear near the posterior root of the meniscus (within 10 mm); 3. Kellgren Lawrence grading of grade III or below; 4. Agree to participate in this study and sign the informed consent form. Exclusion criteria: 1. Patients with combined knee ligament injury requiring ligament reconstruction surgery; 2. Patients with knee joint dislocation (inversion or eversion>8°) or congenital knee joint deformity; 3. complicated with serious primary diseases such as heart, brain, liver, kidney or hematopoietic system; 4. Pregnant and lactating women; 5. Patients with mental illness.

Initially, A total of 40 patients participated in this study, consisting of 17 males and 23 females, with an average age of 22–45 years. All patients underwent either arthroscopic transtibial pullout technique or all-inside meniscus repair surgery.

Patients were divided into two groups based on the surgical method: 20 in the transtibial pullout technique group (including 12 cases of medial meniscus tears and 8 cases of lateral meniscus tears) and 20 in the all-inside repair group (including 11 cases of medial meniscus tears and 9 cases of lateral meniscus tears).

## 3 Surgical techniques

All patients underwent surgery performed by the same surgical team.Patients typically receive general or regional anesthesia (epidural or spinal) to ensure they are pain-free and relaxed during surgery. General anesthesia induces unconsciousness, while regional anesthesia numbs the lower body, based on the patient's condition and surgeon's preference.

### 3.1 Transtibial pullout technique group

Once anesthesia is administered, the patient is positioned supine with the knee flexed at 90°. The surgeon creates anterolateral and anteromedial arthroscopic portals for joint access. Diagnostic arthroscopy is performed to assess the meniscal tear and other joint structures. The torn meniscus root is debrided, removing damaged tissue to expose healthy tissue, and the attachment site on the tibial plateau is prepared.

Under arthroscopic guidance, a guide pin is inserted through the anteromedial tibia to the meniscal root. A reamer then drills a tibial tunnel (6–8 mm in diameter) without damaging surrounding cartilage. A suture-passing device is used to place nonabsorbable sutures through the meniscal root, which are pulled through the tibial tunnel. Multiple sutures may be used to ensure secure fixation.

Tension is applied to reduce the meniscus to its anatomic position, restoring normal biomechanics. The sutures are then secured over a cortical button (Johnson & Johnson Synthes) or anchor (Johnson & Johnson Synthes) on the tibial cortex. A final arthroscopic inspection confirms the meniscus is securely reattached and stable.

**Fig. 2:**
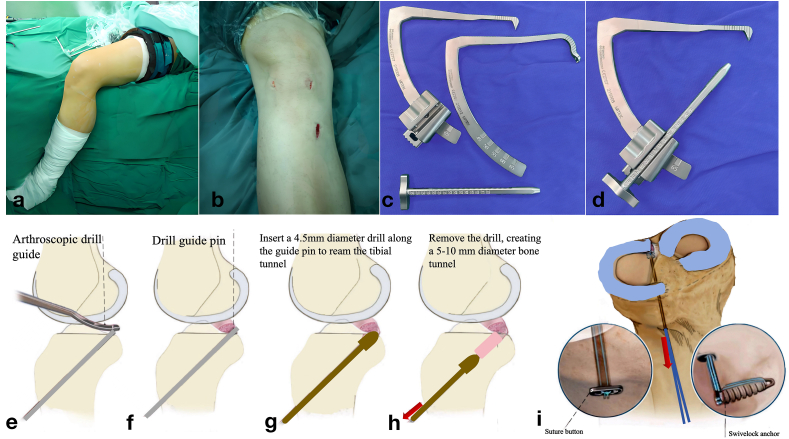
**This figure illustrates the steps involved in a transtibial pullout repair of the medial meniscus posterior root tear. The following steps and components are depicted in the image:** (a) The patient's leg is positioned with the knee flexed and prepared for surgery. The surgical field is covered with sterile drapes, with the leg immobilized using a leg holder.**(b) That** shows the external incision sites on the knee used for arthroscopic instrument entry. The tibial incision is typically located on the anteromedial side of the tibia, approximately 2–3 cm below the tibial plateau. This incision allows for precise insertion of the guide pin, drill, and other instruments while minimizing damage to the surrounding tissues. **(c)** and **(d)** Images of the arthroscopic drill guide and associated instruments used in the procedure. The tools include a calibrated drill guide with adjustable angulation to create an accurate tibial tunnel. **(e)** to **(h)** Illustrations showing the step-by-step process of tibial tunnel creation. **(e)** The arthroscopic drill guide is positioned to identify the ideal tibial entry point. **(f)** A guide pin is inserted into the tibia at the identified location. **(g)** A 4.5 mm diameter drill is used to ream the tibial tunnel along the guide pin. **(h)** The drill is removed, leaving a 5–10 mm diameter bone tunnel for the passage of sutures. **(i)** Final fixation step showing the use of a suture button and a SwiveLock anchor for securing the repaired meniscus root to the tibia. The inset images highlight the suture button (used for secure knotless fixation) or the SwiveLock anchor positioned at the tibial tunnel exit, reinforcing the meniscal repair.

**Fig. 3:**
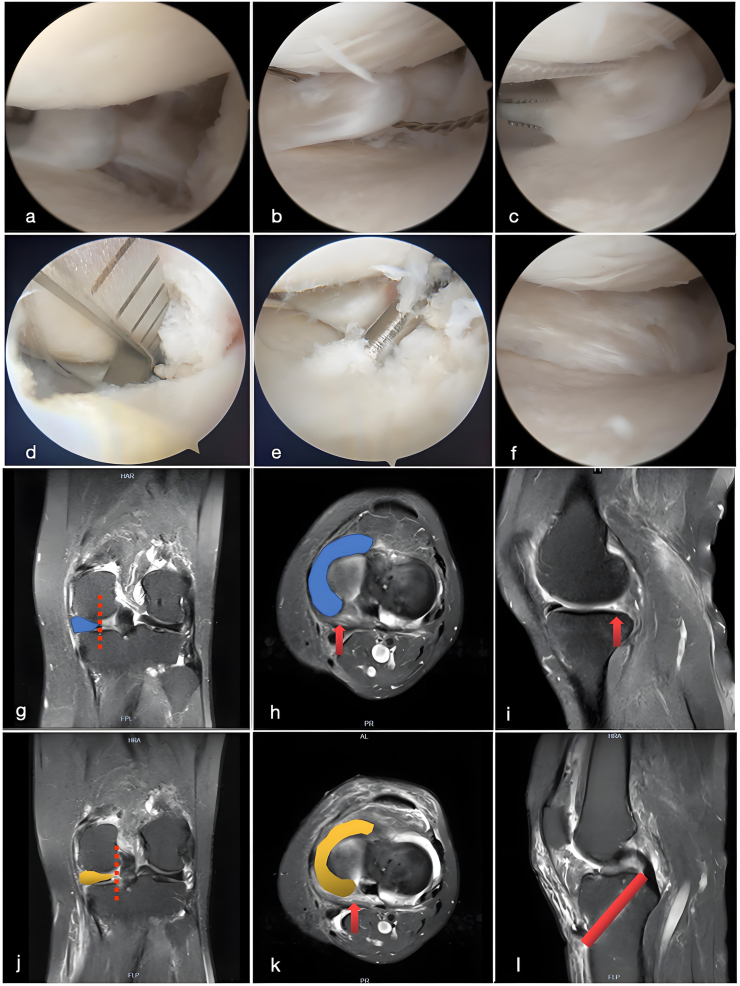
Comprehensive Arthroscopic and MRI Evaluation of Medial Meniscus Posterior Root Tear and Reconstruction Using the Transtibial Pullout Technique. a. Arthroscopic image illustrating a complete tear of the medial meniscus posterior root. The detached root is visualized within the posterior compartment, indicating significant biomechanical compromise of the meniscus; **b.** A suture-passing device is utilized to guide sutures through the torn posterior root. This step is critical for securing the posterior root for subsequent repair, ensuring adequate fixation; **c.** The posterior root is sutured, preparing the torn meniscus for anatomical reattachment. The sutures are precisely placed to allow for secure fixation through the tibial tunnel; **d.** An arthroscopic tibial guide is used to position the tibial tunnel with precision. Accurate tunnel placement is essential for maintaining the correct tension and alignment of the repaired meniscus; **e.** Under arthroscopic guidance, a tibial guide pin exits at the designated location, marking the site where the tibial tunnel will be established. This ensures that the sutures can be pulled through the tunnel to achieve secure fixation of the meniscus; **f.** The sutures passed through the tibial tunnel are pulled out, completing the reconstruction of the posterior root attachment. The arthroscopic image confirms successful reattachment of the posterior root at its anatomical location, restoring meniscal function; **g.** Preoperative coronal MRI highlights the tear in the posterior root of the medial meniscus (red arrow), with lateral displacement of the meniscus body (blue-shaded region). The disruption of the posterior root attachment compromises the stability and load-bearing function of the meniscus; **h.** Preoperative axial MRI further illustrates the extent of the posterior root tear (red arrow) and the lateral displacement of the meniscus (blue-shaded region), consistent with a loss of hoop stress distribution; **i.** Preoperative sagittal MRI shows a clear discontinuity in the posterior root (red arrow), confirming the diagnosis of a medial meniscus posterior root tear; **j.** Postoperative coronal MRI after Transtibial Pullout reconstruction demonstrates restoration of the medial meniscus to its original anatomical position (yellow-shaded region, red dashed line indicating the repaired root). The meniscus is appropriately tensioned and repositioned, indicative of a successful repair; **k.** Postoperative axial MRI confirms the successful reattachment of the posterior root at the anatomical insertion site (yellow-shaded region), with no evidence of displacement or residual gap, ensuring the restoration of meniscal function; **l.** Postoperative sagittal MRI depicts the trajectory of the tibial tunnel established during the Transtibial Pullout technique (red bar). The tunnel provides a secure pathway for suture passage and fixation of the posterior root, facilitating optimal biomechanical restoration.

After surgery, the portals are closed, and the patient is placed in a brace to restrict weight-bearing and knee flexion for several weeks, followed by rehabilitation to restore knee function and mobility (See Fig. 2, Fig. 3).

### 3.2 All-inside repair group

After the patient is positioned supine with the knee flexed at 90° under general or regional anesthesia, the surgeon creates anterolateral and anteromedial arthroscopic portals for access. A diagnostic arthroscopy is performed to assess the tear and joint health.

The torn meniscus edges are then debrided to remove damaged tissue, and the attachment site is prepared for repair. Using a specialized all-inside suture device, nonabsorbable sutures are passed through the meniscus in a vertical or horizontal mattress configuration, securely anchoring the meniscus without the need for a tibial tunnel. The sutures are tensioned to reduce the meniscus to its anatomic position, restoring knee biomechanics.

After confirming stability through arthroscopic inspection, the portals are closed. Postoperatively, the patient is placed in a knee brace to limit movement and prevent weight-bearing for several weeks, followed by a rehabilitation program to restore knee function.

## 4 Postoperative management

Post-surgery, patients receive antibiotics for up to 48 h. The affected limb is then fitted with an adjustable brace for 2 weeks. From weeks 2–6, patients practice partial weight-bearing walking, gradually progressing to full weight-bearing by week 7. By 3 months post-surgery, light jogging can be introduced.

## 5 Evaluation indicators

The study recorded and compared various parameters between the two groups of patients, including surgical time, postoperative mobilization time, hospitalization duration, International Knee Documentation Committee (IKDC) score, Tegner knee activity level score, Lysholm knee score, and the incidence of complications. The IKDC score was used to assess knee discomfort, with higher scores indicating milder symptoms. The Tegner knee activity level score evaluated knee function, where higher scores corresponded to better joint movement capabilities. The Lysholm knee score was used to assess the overall therapeutic outcome, with higher scores reflecting better treatment effectiveness.

## 6 Statistical analysis

The collected data are analyzed with SPSS software (version 25.0). Measurement data are presented as mean ± standard deviation ($\bar{x}$ ± s), and comparisons between the Transtibial pullout technique and All-inside repair groups are made using the Student's t-test. Categorical data are analyzed using the Chi-square test or Fisher's exact test. A p-value of less than 0.05 is considered statistically significant.

## 7 Results

This study compared the outcomes of two surgical techniques for knee injury repair: the Transtibial pullout technique and the All-inside repair method, with 20 patients in each group. The analysis covered demographic characteristics, pre- and postoperative clinical scores, and overall postoperative outcomes (Table 1, Table 2, Table 3).

**Table 1:** Demographic and clinical characteristics of patients undergoing Transtibial pullout technique and All-inside repair surgeries.

Parameters	Transtibial pullout technique group (n = 20)	All-inside repair group (n = 20)	P value
Sex, n, (M/F)	9/11	8/12	
Age, years, mean ± SD	37.65 ± 1.24	38.05 ± 1.34	0.824
BMI, kg/m^2^, mean ± SD	24.11 ± 1.95	24.39 ± 1.24	0.514
Kellgren-Lawrence classification:			
0	0	0	
1	0	0	
2	5	6	0.851
3	15	14	0.714
Operation time, min, mean ± SD	110.15 ± 14.15	102.5 ± 11.41	0.026
Postoperative ambulation time, h, mean ± SD	21.5 ± 5.09	50.55 ± 12.05	0.000
Hospitalization duration, d, mean ± SD	7.45 ± 1.28	6.45 ± 1.32	0.027
Complication:			
Poor wound healing	3	0	
Infection	1	0	
Deep Vein Thrombosis,DVT	0	1	
Posterior root re-tear	0	0	

BMI, Body Mass Index. P-value< 0.05 is considered statistically significant.

**Table 2:** Preoperative and postoperative comparison of IKDC score, Tegner knee activity level score, Lysholm knee score and AROM of knee flexion in Transtibial pullout technique and All-inside repair groups.

Parameters	Preoperative	Postoperative	P value
**Transtibial pullout technique group,n = 20**			
IKDC score, mean ± SD	31.84 ± 3.71	52.82 ± 4.47	0.000
Tegner knee activity level score, mean ± SD	3.69 ± 0.66	7.69 ± 1.19	0.000
Lysholm knee score, mean ± SD	50.39 ± 4.39	84.41 ± 4.31	0.000
AROM of knee flexion, (◦), mean ± SD	46.45 ± 3.78	102.25 ± 7.52	0.000
**All-inside repair group, n = 20**			
IKDC score, mean ± SD	30.31 ± 3.66	46.43 ± 4.81	0.000
Tegner knee activity level score, mean ± SD	3.71 ± 0.66	5.96 ± 0.81	0.000
Lysholm knee score, mean ± SD	51.25 ± 4.41	79.32 ± 4.88	0.000
AROM of knee flexion, (◦), mean ± SD	46.95 ± 5.28	99.85 ± 7.38	0.000

IKDC score, International Knee Documentation Committee score; AROM, Active Range of Motion.

P- value < 0.05 is considered statistically significant.

**Table 3:** Comparison of postoperative outcomes between Transtibial pullout technique group and All-inside repair group.

Parameters	Transtibial pullout technique group (n = 20)	All-inside repair group (n = 20)	P value
**IKDC score, mean±SD**			
Preoperative	31.84 ± 3.71	30.31 ± 3.66	0.119
Postoperative	52.82 ± 4.47	46.43 ± 4.81	0.000
**Tegner knee activity level score, mean±SD**			
Preoperative	3.69 ± 0.66	3.71 ± 0.66	0.932
Postoperative	7.69 ± 1.19	5.96 ± 0.81	0.000
**Lysholm knee score, mean±SD**			
Preoperative	50.39 ± 4.39	51.25 ± 4.41	0.440
Postoperative	84.41 ± 4.31	79.32 ± 4.88	0.001
**AROM of knee flexion, (◦), mean±SD**			
Preoperative	46.45 ± 3.78	46.95 ± 5.28	0.666
Postoperative	102.25 ± 7.52	99.85 ± 7.38	0.240

IKDC score, International Knee Documentation Committee score; AROM, Active Range of Motion.

P-value< 0.05 is considered statistically significant.

### 7.1 Patient demographics and clinical characteristics

Table 1 shows no statistically significant differences between the groups regarding demographic characteristics such as sex distribution (Transtibial: 9 males, 11 females; All-inside: 8 males, 12 females), age (Transtibial: 37.65 ± 1.24 years; All-inside: 38.05 ± 1.34 years, p = 0.824), and body mass index (BMI) (Transtibial: 24.11 ± 1.95 kg/m^2^; All-inside: 24.39 ± 1.24 kg/m^2^, p = 0.514). The severity of knee osteoarthritis, assessed using the Kellgren-Lawrence classification, was also comparable, with no significant differences in grades 0 to 3 between the groups (p > 0.05).

However, there were significant differences in operative time, postoperative ambulation, and hospital stay. The Transtibial group had a longer mean operative time (110.15 ± 14.15 min) compared to the All-inside group (102.5 ± 11.41 min, p = 0.026). In contrast, the Transtibial group demonstrated significantly faster postoperative ambulation (21.5 ± 5.09 h) than the All-inside group (50.55 ± 12.05 h, p < 0.001), indicating a quicker recovery. The Transtibial group also had a slightly longer hospitalization duration (7.45 ± 1.28 days) compared to the All-inside group (6.45 ± 1.32 days, p = 0.027). Complications included three cases of poor wound healing and one infection in the Transtibial group, and one case of deep vein thrombosis (DVT) in the All-inside group, with no statistically significant differences between them.

### 7.2 Preoperative and postoperative functional assessment

**Fig. 4:**
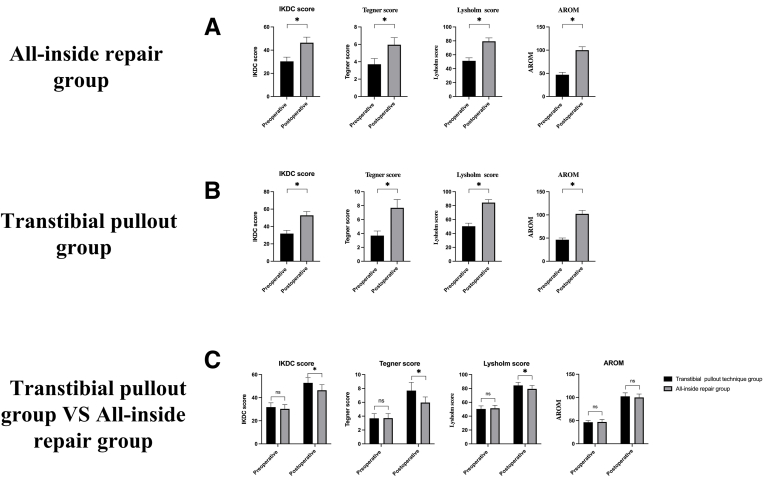
Comparative Analysis of Preoperative and Postoperative Efficacy Indicators in the All-Inside Repair Group, Transtibial Pullout Group, and Transtibial Pullout vs. All-Inside Repair Groups. Comparative analysis of preoperative and postoperative outcomes in the All-Inside Repair group and the Transtibial Pullout group. (A) The first bar graph illustrates the parameter changes for the All-Inside Repair group, with significant improvements observed postoperatively compared to preoperative values; (B) The second bar graph shows the pre- and postoperative results for the Transtibial Pullout group, indicating notable alterations following the surgical intervention; (C) The third bar graph compares the degree of change between the two groups across the different time points, emphasizing the differential effects of the repair techniques. Error bars represent standard deviations, and statistical significance is denoted by asterisks (p < 0.05), indicating meaningful differences in certain parameters between groups.

Table 2 Demonstrates significant improvements in knee function across both groups based on four parameters: the International Knee Documentation Committee (IKDC) score, Tegner knee activity level score, Lysholm knee score, and Active Range of Motion (AROM) of knee flexion (p < 0.001 for all comparisons) (See Fig. 4).

**IKDC Score:** The Transtibial group showed a more pronounced increase in the IKDC score, from 31.84 ± 3.71 preoperatively to 52.82 ± 4.47 postoperatively. Similarly, the All-inside group improved from 30.31 ± 3.66 to 46.43 ± 4.81.

**Tegner Knee Activity Level Score:** A significant improvement was noted in the Transtibial group, rising from 3.69 ± 0.66 to 7.69 ± 1.19, compared to an increase from 3.71 ± 0.66 to 5.96 ± 0.81 in the All-inside group.

**Lysholm Knee Score:** Both groups exhibited substantial gains, with the Transtibial group increasing from 50.39 ± 4.39 to 84.41 ± 4.31, while the All-inside group improved from 51.25 ± 4.41 to 79.32 ± 4.88.

**AROM of Knee Flexion:** Both groups experienced significant enhancements in AROM, with the Transtibial group achieving a slightly higher postoperative mean (102.25 ± 7.52°) compared to the All-inside group (99.85 ± 7.38°).

### 7.3 Complications analysis

Postoperative complications varied between the groups. In the Transtibial group, three cases of poor wound healing occurred at the tibial incision site, likely due to intraoperative mishandling or skin burns from the drill. These wounds eventually healed after multiple dressing changes. Additionally, one case of infection occurred in this group, involving a patient with a history of diabetes and poorly controlled blood sugar levels; the infection was successfully treated with appropriate antibiotics (Table 1).

In the All-inside group, only one complication was reported, a case of DVT that developed due to limited postoperative mobility resulting from joint pain. This condition resolved following standard anticoagulant therapy. Importantly, neither group experienced re-tears of the medial meniscus posterior root during the follow-up period, indicating that both surgical techniques were effective and reliable treatment options (Table 1).

### 7.4 Comparative postoperative outcomes

Table 3 Provides a direct comparison of the postoperative outcomes. The Transtibial group achieved higher postoperative IKDC scores (52.82 ± 4.47 vs 46.43 ± 4.81, p < 0.001) and Tegner knee activity level scores (7.69 ± 1.19 vs 5.96 ± 0.81, p < 0.001) compared to the All-inside group, indicating better functional recovery and greater knee activity. Additionally, the Lysholm score was significantly higher in the Transtibial group postoperatively (84.41 ± 4.31 vs 79.32 ± 4.88, p = 0.001), suggesting more significant symptomatic relief. Although both groups showed substantial improvements in AROM, the difference in postoperative AROM between them was not statistically significant (p = 0.240).

The findings suggest that while both surgical techniques are effective in enhancing knee function following medial meniscus posterior root tears, the Transtibial pullout technique may provide faster recovery, superior functional outcomes, and higher patient-reported knee activity levels compared to the All-inside repair method. However, the slightly higher complication rate and longer operative time associated with the Transtibial technique should be considered during surgical planning. Both techniques demonstrated reliable repair outcomes, with no re-tears observed during follow-up, confirming their efficacy in treating this condition (See Fig. 4).

## 8 Discussion

This study compared the efficacy of the arthroscopic Transtibial pullout technique and the All-inside repair method for treating medial meniscus posterior root tears (MMPRTs), focusing on clinical outcomes, functional recovery, and complication rates. The findings indicate that while both techniques are effective, each has distinct advantages and limitations that can influence surgical decision-making.

### 8.1 Clinical and functional outcomes

The results showed that both surgical methods significantly improved knee function, as evidenced by increased International Knee Documentation Committee (IKDC) scores, Tegner knee activity level scores, and Lysholm knee scores in both groups.^12^, ^13^, ^14^ However, the Transtibial pullout technique consistently yielded superior postoperative outcomes compared to the All-inside repair.^5^ Patients in the Transtibial group demonstrated higher IKDC and Tegner scores, suggesting better functional recovery and enhanced activity levels.^7^^,^^12^ These findings are consistent with previous studies that highlighted the biomechanical benefits of Transtibial fixation, such as stronger anchorage and more stable meniscal root restoration, which contribute to superior clinical outcomes.^8^^,^^15^

The greater symptomatic relief observed in the Transtibial group, as reflected by the significantly higher Lysholm scores, supports the technique's effectiveness in alleviating pain and improving quality of life.^14^ Additionally, although both groups achieved significant gains in the active range of motion (AROM) of knee flexion, the differences between the techniques were not statistically significant.^16^ This suggests that while both approaches facilitate improved knee mobility, the Transtibial technique may offer a slight advantage in restoring normal joint biomechanics.^6^

### 8.2 Complications and safety considerations

The complication analysis revealed a higher rate of adverse events in the Transtibial group, including three cases of poor wound healing and one case of infection, compared to a single case of deep vein thrombosis (DVT) in the All-inside group.^17^^,^^18^ The wound healing issues in the Transtibial group were attributed to potential intraoperative factors, such as skin burns from the drill.^19^^,^^20^ Proper surgical technique and intraoperative precautions are crucial to minimize such risks.^19^ The infection occurred in a patient with diabetes, highlighting the need for meticulous perioperative care and optimal glycemic control in patients with comorbid conditions.^21^

The DVT case in the All-inside group was associated with limited postoperative mobility due to joint pain.^19^ This finding emphasizes the importance of early mobilization and DVT prophylaxis in postoperative care protocols for all patients undergoing knee surgery.^20^ Importantly, no cases of re-tear were reported in either group during the follow-up period, indicating that both surgical techniques are reliable for repairing MMPRTs.^12^

### 8.3 Surgical technique selection

The choice between the Transtibial pullout technique and the All-inside repair method should consider individual patient factors, surgical goals, and the potential risks associated with each approach.^8^ The Transtibial technique offers stronger biomechanical fixation, making it well-suited for complex or larger root tears requiring robust stability.^7^ However, the increased risk of wound-related complications and slightly longer operative time may be limiting factors in certain patient populations.^18^

Conversely, the All-inside repair is less invasive, avoiding the creation of a tibial tunnel, which reduces surgical trauma and operative time.^10^ This approach may be advantageous for patients who are at higher risk for complications associated with more extensive surgical interventions.^9^ However, concerns remain about the fixation strength achieved with the All-inside method, particularly in cases involving more severe tears or poor tissue quality.^11^

This study has some limitations that should be considered when interpreting the results. The relatively small sample size may limit the generalizability of the findings.^13^ Additionally, the follow-up duration was not sufficient to assess long-term outcomes such as osteoarthritis progression or the durability of meniscal repair.^6^ Future research should focus on larger, multi-center studies with extended follow-up periods to confirm these results and provide more comprehensive guidance for clinical practice.^7^ Furthermore, evaluating the impact of rehabilitation protocols on recovery outcomes could help optimize postoperative management.^5^

## 9 Conclusion

Overall, both the Transtibial pullout technique and the All-inside repair method effectively restore knee function in patients with MMPRTs, with the Transtibial technique demonstrating superior functional recovery. The choice of surgical approach should be tailored to the patient's specific needs, considering factors such as tear complexity, comorbid conditions, and potential risks. By understanding the strengths and limitations of each technique, clinicians can make more informed decisions to achieve the best possible outcomes for their patients.

## CRediT authorship contribution statement

**Jun Li:** Conceptualization, Methodology, Funding acquisition, Software, Investigation, Formal analysis, Writing – original draft. **Pengfei Shen:** Data curation, Writing – original draft. **Tao Zou:** Supervision, Resources, Software, Validation. **Wen Min:** Visualization, Investigation. **Yuxing Qu:** Visualization, Investigation. **Zikang Xie:** Conceptualization, Supervision, Resources, Writing – review & editing. **Chengjian Wei:** Visualization, Writing – review & editing.

## Ethics approval and consent to participate

This study was approved by the Ethics Committee of the Changzhou Hospital of Traditional Chinese Medicine.

## Consent for publication

Not applicable.

## Availability of data and materials

The datasets used and/or analyzed during the current study are available from the corresponding author upon reasonable request.

## Studies in humans and animals

We expressly declare that we comply with all relevant regulations, including the ARRIVE guidelines, the U.K. Animals (Scientific Procedures) Act, 1986 and associated guidelines, EU Directive 2010/63/EU for animal experiments, and the National Research Council's Guide for the Care and Use of Laboratory Animals.

## Funding statement

This study was funded by the 10.13039/100018696Changzhou Municipal Health Commission Science and Technology Research Project, No. ZD202222, with a grant amount of 100000 yuan. The design, data collection, and analysis process of this study remain independent and not influenced by any sponsor. We look forward to this research providing new ideas and directions for the development of meniscus treatment.

## Conflict of interest

We declare that we have no financial or personal relationships with individuals or organizations that could have inappropriately influenced the work reported in this paper. All sources of funding for this research are disclosed, and we affirm that the content is unbiased and free from any conflicts of interest.
